# In Search of Genetic Agency: On the Uses and Actionability of Direct‐to‐Consumer Genetic Test Results in Biohacking Practices

**DOI:** 10.1111/1467-9566.70237

**Published:** 2026-07-14

**Authors:** Carsten Stage

**Affiliations:** ^1^ School of Communication and Culture Aarhus University Aarhus Denmark

**Keywords:** actionability, biohacking, direct‐to‐consumer genetic test (DTC GT), epigenetics, milieu

## Abstract

The article examines why and with what consequences biohacking individuals use direct‐to‐consumer genetic tests (DTC GTs). Based on thematic coding of 15 semi‐structured interviews, it argues that these users generally see themselves as capable of generating actionable knowledge to optimise future health by manipulating their ‘milieu’. DTC GTs are integrated into this perspective as a knowledge technology for prevention and optimisation. However, informants often find it difficult to translate DTC test results into concrete action and the study identifies their different strategies for making this type of knowledge actionable. These include monitoring risk conditions, changing or reinforcing existing lifestyle practices, engaging in trial‐and‐error experimentation or postponing action whilst awaiting more advanced knowledge. The article furthermore outlines the relational dilemmas experienced by the informants as a type of consequence of taking DTC GTs. Overall, the article shows the various ways that a highly knowledge‐oriented user group navigates DTC test results by relying on an ‘epigenetic imaginary’ that reframes genetic risk as open to intervention, but also how it struggles to close the gap between test results and the health‐optimising actions that are so central for biohacking practices.

## 1 Introduction

Since the launch and spread of direct‐to‐consumer genetic testing (DTC GT) in the 2000s, millions of people have purchased a DNA home testing kit in the hope of learning more about their health or ancestry (Regalado 2019; Saukko 2017). This paper explores how health‐related DTC GTs are used—and struggled with—by a group of informants who self‐identify as involved in biohacking activities. Biohacking is premised on a ‘central belief in intervention into the very living matter of the body’ (Cozza et al. 2022, 4) and often also on an interest in how genetic knowledge can be used to slow down ageing, increase human performance and prevent health problems. For that reason, many biohackers have taken up DTC GTs alongside a range of other technologies and health‐related practices. Based on 15 interviews with people who understand themselves as involved in biohacking practices and who have taken one or more health‐related DTC GTs, the paper answers the following RQ:

How are direct‐to‐consumer genetic test results used and rendered actionable in biohacking practices?

The more specific analytical focus of the article will be on how the informants try to deploy or translate DTC GT test results in the attempt to improve their current and future health status. They do so by tapping into an ‘epigenetic imaginary’ that frames genetic risks as manipulable by environmental factors. This, however, also produces a problem for the informants: How to turn test results into actions that manipulate the milieu in meaningful and impactful ways? What can a biohacker do if she wants to decrease a certain type of genetic risk? The informants in other words struggle with how to bridge the gap between test results and actions with perceived positive (epi)genetic consequences and the analysis outlines the informants' different strategies for turning knowledge provided by DTC GTs into some sort of action.

The reason for studying DTC GT in the context of biohacking is to investigate the underexplored question of how some users make these test results actionable in their own ways outside health contexts and thus to question the notion that DTC GTs most often do not lead to changes (McGowan et al. 2010; Nolan and Ormondroyd 2023). In that way the study of biohacking practices provides new knowledge about the use of DTC genetic testing by offering a better understanding of some of the various action potentials of such tests from a user perspective.

## 2 Existing Research on Biohacking and DTC GT

### 2.1 Biohacking the Milieu

Biohacking originally referred to a ‘movement of amateurs conducting life sciences outside of traditional professional settings such as university and corporate labs’ (Delfanti 2012, 163). For that reason, the movement—which consisted of both biologists, ‘computer geeks’ and artists—was also described as ‘garage biology’ or ‘DIY biology’ and based on a creative desire to tinker with biological processes to optimise health and the body (Delfanti 2012).

The term biohacking can be traced back to a *Washington Post* article from 1988, but the organisational foundation of the movement is often attributed to Boston in 2008, where a group of biohackers assembled through mailing lists and an internet forum (Meyer and Vergnaud 2020). Today biohackers are still often organised in and through large dedicated online groups, which can be transnational, like ‘Biohackers (GLOBAL)’ (17,600 Facebook members) or national in scope, like ‘Danish Biohacker Community’ (3600 Facebook members).

Biohackers often engage with a wide array of body‐technological practices besides DTC GT, for example, ‘Gene editing and splicing devices, microchip implantation, cryo‐tanks and infrared saunas to diet and fitness regimes, nootropics and enzyme supplements’ (Cozza et al. 2022, 4). This is, however, done in different ways and with different goals. Existing research has distinguished between three archetypes of biohackers: (1) ‘Citizen scientists’ (e.g., Zayner) that ‘design and deploy their own equipment, use their own bodies as sites of experimentation and open‐source their results’ in order to democratise and de‐institutionalise the life sciences (Rifai 2022, 4). (2) ‘Cyborgs’ (e.g., Harbisson, Warwick, Spence), who experiment with the boundaries between the human body and technologies, for instance through the use of body implants. (3) ‘Superhumans’ (e.g., Asprey, Ferriss) that aim at optimising the body and decreasing decay and biological age (Rifai 2022). This quest for a superhuman ability to slow down ageing makes the difference between biohacking and dedicated health activities, self‐tracking and longevity hard to define. Biohacking activities in that way overlap with an increasingly mainstreamed preoccupation with food supplements, nutrition experiments and tracking gadgets.

Biohacking is characterised by a biopolitical quest for individual control through knowledge about and creative manipulation of the body and its environment. Here prevention and present/future vitality become the central goals of biopolitical (self)interventions (Rose 2006, 18). Thomas Lemke has focused on how knowledge about genetic risk, but also material arrangements, can play an important role in these attempts to control health futures. According to Lemke, this kind of control work is dependent on ‘the production of knowledge that seeks to transform uncertainties into risks and probabilities’, because risk is more actionable than uncertainty (Lemke 2021, 132). By identifying individual risks, the subject moves from inhabiting an unpredictable world to being an agent that can actively engage with specific problems and thus potentially shape its world and future.

Lemke argues that this type of practice relies on a specific understanding of the environment—or ‘milieu’—that the subject occupies. The milieu is not only seen as a container or a set of external stimuli, but rather as a kind of medium that the subject tries to actively interact with, manipulate or modulate in order to produce a certain trajectory from the present into a desired version of the future: ‘The milieu defines a spatio‐temporal arrangement of a hybrid ensemble of material entities mobilised to control future trajectories and to achieve specific goals (…)’ (Lemke 2021, 131). In that way, a sense of deep connections between the body and its material milieu is intertwined with a desire for individual and temporal control. Knowledge of genetic risk can be perceived as involved in these attempts ‘to produce, if seemingly paradoxically, a new form of autonomous subjectivity’ (Lemke 2004, 558) through the subject's active engagements with its milieu as a medium of control. Biohackers' use of DTC GTs is an example of how genetic knowledge production is integrated in the quest for manipulating spatio‐temporal arrangements, but also of how the bridge between knowledge and actions is not always easy to cross.

### 2.2 Direct‐to‐Consumer Genetic Testing

DTC GTs provide consumers with insight into their risk of developing conditions such as Alzheimer's disease, cancer and Parkinson's disease. Results are most often received and interpreted without medical intermediaries or counselling. DTC GT companies frame genetic self‐testing as easy, enjoyable and as a technology with a nonpaternalistic aura (Saukko 2018). However, the DTC genetic health tests have also spurred controversies due to the commercialisation of genetic testing, the lack of data control and clinical guidance, the risk of overdiagnosis, excessive medicalisation and the questionable medical validity of the results.

Most DTC GTs offer SNP (Single Nucleotide Polymorphisms) genotyping that focuses on a limited number of already specified genetic variations, but whole genome sequencing (WGS) that creates an overview of the entire genome and therefore also potentially rarer dispositions, is also available on the DTC market. Although this study primarily focuses on the use of genetic tests focused on genetic risk markers, so‐called ‘epigenetic tests’ (like My DNAge) are becoming more prominent in biohacking communities and are also mentioned by some informants in this study. Although traditional DTC GTs focus on identifying polygenic risk factors (cf. the risk of getting certain diseases), epigenetic tests often measure methylation processes that regulate DNA expression. This type of test offers (a medically contested) assessment of the biological age of the tester, which can be different from chronological age, but also identifies life‐style activities that are said to be able to improve biological age.

Biohackers' interest in (genetic) knowledge as a road to health agency implies a certain approach to, or imaginary of (Franklin 2000; Van Dijck 1998), genes and genetic testing and to where this knowledge can actually be put to use and influence the life of the tester. The intertwinement of the biohacker's desire for action and engagement with genetic knowledge production thus seem to prioritise an epigenetic understanding of genetic expression as manipulable through environmental factors instead of earlier models of genetic determinism (Bull 2019; Saukko 2017). Following this, the article uses the term ‘epigenetics’ both to refer to a particular type of test, but also to describe the informants' broader understanding of genetics as a field open to human action and manipulation. Exactly how biohackers' desire for action tackles, translates or acts on DTC genetic test results have, however, not been explored. Research on biohacking and genetics has instead primarily focused on genome editing/CRISPR (Martin et al. 2020), the figure of the (bio)cyborg or artificial chimera (Pio‐Lopez 2021) and on biohacking as a form of citizen science (Guerrini et al. 2022).

Previous research into DTC GT has concentrated on topics such as ancestry and kinship (Horowitz et al. 2019; Gregory 2019), commercial marketing (Williams‐Jones 2006), digital communication (Harris et al. 2013), privacy and regulation (Curnutte 2017; Raz et al. 2020) and the perspectives of and implications for health care professionals (Markens 2013; Van Steijvoort et al. 2025). Studies of users have suggested a diversity of motivations for engaging with DTC GTs, including genetic curiosity, interest in ancestry and the pursuit of lifestyle guidance (Gerdes et al. 2021; Roberts et al. 2017). Early adopters are often highly literate in genetics and risk assessment, yet the behavioural impact of such tests have been shown to be minimal (McGowan et al. 2010), although many users also express a desire for using test results to influence their health behaviours (Roberts et al. 2017). Nolan and Ormindroyd, in a review of the consequences of DTC GTs for consumers, conclude that consumers ‘are broadly satisfied with and perceive personal utility from DTC‐GT, although many do not share results with a HCP or act on results’ (Nolan and Ormondroyd 2023, 18). Simultaneously, a new epigenetic discourse is taking shape around DTC GTs, wherein metaphors shift from the gene as a deterministic ‘book of life’ to more dynamic or agential metaphors (Saukko 2017). In that way DTC GTs are increasingly understood as linked to ideas of genetic agency and as tools for informing lifestyle choices, but paradoxically they are rarely acted on by actual test users due to difficulties with closing the gap between test results and action.

DTC GTs have also been shown to produce a range of communicative, relational and privacy‐orientated dilemmas in terms of how to share information about personal test results with potentially implied family members and when to encourage further testing of relatives (Middleton 2012; Sleiman et al. 2025; Matranga 2020). These findings have many similarities in common with research on the complex genetic bonds and communicative challenges produced through clinical genetic testing (Svendsen 2002; Kenen et al. 2003; Arribas‐Ayllon et al. 2008).

The article contributes to the study of user perspectives on DTC GT by outlining the ways that a particular user group (struggle to) act on and with test results. In that way the article sheds light on the complex ‘actionability work’ involved in using DTC GTs. The study also contributes to studies of biohacking with its focus on their deployment of genetic knowledge generated outside health institutions—but also by integrating a particular conceptual framework (the milieu) in exploring this group's approach to their embodied health activities.

## 3 Method

The article's research design is constructionist in the sense that it focuses on the informants' own interpretative understanding of their practices, not on how these practices are actually played out or on the medical correctness of how they understand genetics (Creswell 2014). The study draws on semi‐structured interviews with 15 individuals (5 identified as women and 10 as men) aged between 30 and 63. The sample is composed of 11 Danes and four non‐Danes (from Poland, the United Kingdom and Hungary), all with a high level of education and professional backgrounds spanning health, art, IT, law and business. Interviews were conducted between February and April 2024. When quoted, informants are identified by their self‐identified gender (F or M) and their age.

Participants were recruited through a range of online biohacking and DTC GT communities, including the ‘Danish Biohacker Community’, ‘23andMe Forum’, ‘Biohackers (GLOBAL)’ on Facebook and the subreddit ‘r/Biohacking’. Informants were chosen due to their accessibility and their willingness to be interviewed about how DTC GTs are experienced from a biohacking perspective. The main criteria for inclusion were that people understood themselves as biohackers or as persons involved in biohacking activities and that they had taken a health‐related DTC GT.

The reason for focusing on biohackers was due to their high level of interest in health knowledge and in translating knowledge into action. The selection of informants—and the analytical approach—is not aimed at generalisation but instead enables an in‐depth understanding and theorisation of how informants with a particular biohacking mindset and practice engage with genetic knowledge provided by DTC GTs.

The interviews were conducted either face‐to‐face or online, depending on the participants' geographical location and preference. Each interview lasted approximately 60 min and was recorded and transcribed in full. The interview guide was thematically structured to elicit reflections on participants' understanding and history of biohacking, their motivations for using DTC GTs and experience of the testing process, their interpretations of genetics and test results and how their (biohacking) practices were affected by the results.

All participants gave written informed consent, were told that they could cancel participation at any point and offered the chance to read transcripts (four decided to do so but had no substantial comments). The interview and transcription process was supported by research assistant Katrine Krøjgaard. The material was inductively coded and guided by the topics that structured the interview guide (Braun and Clarke 2021; Braun and Clarke 2006). This article will focus on the themes generated to interpret the informants' motivation for using DTC GTs, their understanding of genetics and DTC genetic testing in relation to biohacking and on the different ways that informants acted on test results.

## 4 Analysis

The analysis is structured in two parts. The first deploys the concept of the milieu to describe the informants' understanding of genetics and their general desire for knowledge‐based and often preventive action. The second part explores how this desire for translating knowledge into concrete actions is played out in relation to receiving DTC test results.

### 4.1 Constructing (Epi)genetic Agency

The informants perceive themselves as individuals engaged in biohacking activities, but with different levels of investment. Some of them are key and public figures in the movement, offer talks on biohacking and advise other people on biohacking issues; others are members of a few online groups where they find inspiration and knowledge. Furthermore, some informants see it as a crucial part of their identity, whereas others perceive themselves as ‘light’ biohackers or as ‘doing biohacking’ without explicitly using it as part of their self‐presentation. Several informants even describe that the biohacker category can be a little stigmatising and that they are not (cyborg) biohackers interested in body implants.

Across the interviews the informants mention an array of practices that they themselves associate with biohacking, for instance systematic use of a range of food supplements and methyl donors, cold water baths, various tests, blood donation, light exposure, breathing routines and patterns, meditation, low tox practices, micro‐dosing, use of anti‐radiation technology and intermittent fasting and fasting mimicking diets. Many of the informants are not engaged in highly avantgarde health‐technological practices, but they are all deeply engaged in constantly finding new knowledge to help them optimise their health and they frame this knowledge work as linked to biohacking as a practice.

The informants also share a range of key assumptions regarding knowledge, genetics and healthcare that are important as a context for understanding their engagement with DTC GTs.

#### 4.1.1 Knowledge as Power, Prevention and Control

Previous research has identified fear and concern as central emotions regarding the future when living with an identified genetic predisposition (Petersen et al. 2014; Huniche 2003; Youssufi and Huniche 2017). These feelings are not prevalent in the material as the informants seem to link the process of getting knowledge about their health risks as highly motivational, reassuring, connected to a sense of control and in many ways affectively undramatic.

A female informant describes that with a biohacking mindset ‘you know that the risks you're informed about are things you can do something about, so you become much more enthusiastic’ (F63), whereas a male informant describes genetic knowledge production as linked to a kind of affective calmness: ‘I calmed myself down with knowledge and realised that there was plenty I could do to prevent these things’ (M43). A female informant also describes how knowledge about genetic risk is ‘in no way negative. Maybe just a motivational factor in relation to doing strength training, then I just think it's even more important for me to do it, right? So no, I really haven't had any negative feelings about it, at all’ (F56). Some of the words used for the informants' desired outcome of biohacking and genetic testing are ‘control’ or ‘power’: ‘You want to know yourself on an individual basis rather than just be … taken care of by the health care system. You want to take charge, be in control’ (M35). Or: ‘I believe that knowledge is power’ (F45).

The informants generally use DTC GTs—alongside a range of other technologies and practices—to try to turn uncertainty into more tangible and actionable risk assessments. Instead of guessing about potential dangers lying ahead, the informants aim at producing a milieu where certain practices are identified as more likely to support their health than others. In that way the genetic tests become what Lemke describes as a ‘resilience technology’ (Lemke 2021, 176) that are used to support the informants in developing targeted forms of optimisation or prevention. In temporal terms the informants thus seem to link DTC genetic testing to notions of ‘getting ahead’ (M30), ‘pre‐emption’ (M48), ‘pro‐activity’ (M30) and ‘prevention’ (M30).

The informants acknowledge the inevitable arrival of death but want to live as vital a life as possible and avoid suffering. A male informant describes that his goal is ‘to die with your boots on at a hundred years old, being fresh and active and accidentally falling off a cliff, right?’ (M43), whereas a female informant states that she wants to ‘live a long life, but to be healthy, fit and happy the whole way through. And then, when it's time to die, I'll just lie down and pass away and that's that. That's essentially my goal’ (F45). In other words, the reason for seeking knowledge is both to postpone death and to eradicate decay and suffering during life as much as possible.

#### 4.1.2 An Epigenetic Imaginary

A vast majority describe how they understand the genome as a stable, but with effects that can be affected by environmental factors shaping gene expression. Some informants explicitly use the term ‘epigenetics’ to describe this line of thinking:

- ‘When I take a genetic test, it's based on the genetics I've inherited. But the environment in which the genes develop—that's through epigenetics, that's environment. It's a combination of environment and inheritance, as I see it’ (M40).
- ‘Well, the genes themselves—you can't change them, so they're a given condition. But then there's epigenetics, this idea of whether something is switched on or off. And that's something you can influence through your lifestyle’ (F45).
- ‘And that's why I think your genetics—depending on who you ask—only about 10 or 20 percent of it is actually fixed. The rest is something you essentially activate through your lifestyle, it is called epigenetics. What I'm much more focused on is which genes—put simply—are switched on and off’ (M43).

The desire for life‐enhancing control is in that way articulated by the informants as intertwined with what could be called an ‘epigenetic imaginary’, which assumes that the personal genome cannot be changed in its structure, but that genes can be activated or deactivated in terms of their consequences. An informant describes how this type of pragmatic and action‐orientated thinking is at the very core of biohacking practices, which, according to him, is all about taking ‘control over both the inner and outer environment’ based on the premise that ‘the way we move and what we do in our external environment, as well as what we put into our bodies, of course, also affects our internal environment’ (M43). In that way the informant approaches himself as being part of a milieu or a spatio‐temporal arrangement, that can be governed to obtain internal health or bodily goals. His surroundings can be organised and steered to move his body into the future in desired ways. He continues:

Lifestyle, diet, exercise, air, water, oxygen, everything. Everything you move in and do. Everything you consume. Your thoughts. There are plenty of studies that suggest that with the power of your thoughts, you can turn genes on and off.

(M43)

As Saukko has pointed out the turn to epigenetics has also implied a shift in genetic metaphors from deterministic ideas of genes as a ‘code’, ‘blueprint’ or ‘book of life’ to more action‐orientated metaphors of genes as ‘switches’ that can be turned on or off or as ‘instruments’ that can be played in different ways: ‘I've always had this approach to it, that it's just my genetics. I choose how I want to play the piano I've been given’ (F45).

These metaphors reflect the shift from ‘genetic determinism’ to a more contextual and lifestyle‐orientated genetic imaginary (Franklin 2000; Bull 2019). This helps explain why a negative genetic risk assessment is not necessarily a dreaded verdict for the informants. It is rather understood as a chance to positively shape the body by re‐arranging its milieu. In that way epigenetics is used by informants to undergird their sense of agency in terms of being able to affect their health through external stimulation, but, as we shall see, this also produces a tension as it is not always clear what actually turns genes on/off or what can be done about the genetic dispositions revealed by the test.

#### 4.1.3 The Flaws of Healthcare

The informants also share a critical‐reformist and sometimes even disappointed or angry approach to the traditional healthcare system. This can be based on general reflections on how genetics and personal medicine ought to be the future of a more effective healthcare system that prevents illness instead of only treating symptoms. One informant for instance argues that all citizens should get a genetic test as this would lead to effectivization and prevention: ‘I simply cannot understand why we are not doing it. It would be cheaper; it would be better. It would—I genuinely think we would have a better healthcare system’ (F37). In that way some informants also describe themselves as an avantgarde that has understood and started to use the potentials of genetic health technologies in contrast to a healthcare system lagging behind.

Control can also become a goal due to less‐than‐optimal experiences with the healthcare system, where the informants felt that they were not cared for and therefore needed to be more proactive. Some informants, in other words, articulate a fair amount of systemic disappointment:

(…) I've spent many years being disappointed – at least in retrospect – disappointed that I was so naive as to think my doctor had control over my health. He didn't have control over it at all. My doctor managed my symptoms, but he didn't do anything to prevent me from getting sick.

(M43)

When the informants describe their engagement with the tests, a will to take on personal health responsibilities and an interest in prevention is conflated with the idea that knowledge about health risks produces an increased ability to act instead of being pushed around by unpredictable systems:

Biohacking might be where you're at the forefront, but generally, there's a trend of taking a bit more responsibility for your own health – out of interest – and, you could say, out of necessity as well. We don't exactly have a healthcare system that is particularly focused on prevention. So, if you're the type who wants to be proactive, you must take matters into your own hands. And that's where DNA tests fit in well.

(M59)

The backdrop for this motivation to be in control through biohacking is the desire to either stay healthy or to address and combat already experienced conditions and pain. A female informant, who has struggled with various conditions and often faced a system unable to help her, explains: ‘(…) if you're down where it's just about survival, then it's just the body making noise and you'd like it to shut up’ (F63). Here the wish to be in control is linked to restoring health as an experience of ‘the silence of the organs’ (Canguilhem 1991, 101) or of living with a body that stops being a disturbance.

### 4.2 How to Act on and With DTC Test Results?

The informants integrate the use of DTC GTs in an understanding of themselves as milieu‐shaping individuals but often also struggle to figure out if and how the assessment of genetic risk can be used and instrumentalised in terms of manipulating their milieu. A few informants conclude that this has been difficult and that the results or data produced by the tests are therefore not really actionable yet. A majority, however, still describes different types of action motivated by receiving test results. These actions do not necessarily focus on lifestyle changes but can have a range of different implications. Some are very directly linked to the content of the results and on changing health behaviour, whereas others seem to be motivated more by attempts to supplement or replace test results with other types of knowledge. The analysis seeks to outline the different ways that test results become part of the actions and activities of the informants.

#### 4.2.1 Self‐Surveillance and Demedicalisation

A type of action relates to the healthcare system. One informant feels that his experience of his own health has been confirmed by the test: ‘It has had the effect that it can confirm what you experience, validate what you experience. Then you can say, okay, I know I'm not crazy. Maybe the doctor is, but at least I'm not crazy’ (M44). This implies that the informant thinks that he can contact healthcare with a stronger confidence in his own lived experience. A perhaps more tangible consequence relates to informants that initiate various types of health surveillance to ensure that identified risks do not develop into a diagnosis. A female informant has identified a risk of cancer through a DTC GT, which has led her to pay for a personal monitoring programme to be able to postpone and act on the disease before it becomes an actual condition:

And when I turn 40, I will start annual colonoscopies, meaning a camera up the rectum or a camera all the way down the throat. That begins… and it's a protocol I've decided on myself. It's something I'll pay for myself; it's not something provided by the public healthcare system. This means I'm spending money and resources on this based on the knowledge I've gained and it's not something the public system can offer, even though my risk of gastrointestinal cancer is three times higher than that of an average Dane.

(F37)

For this informant, DTC genetic knowledge production is acted on through preventive health monitoring that allows her to react quickly if the disease risk develops further. Another informant describes how results made him contact a cardiologist as the test ‘did seem to indicate some points where I had a higher risk. So, in this sense I could use it’ (M48). Another user integrates risk knowledge in other forms of testing activities: ‘But I'll bear it in mind and if I start seeing cardiac markers on blood tests something like this, then yeah, I'll go okay, I probably have to take this more seriously’ (M34). These different examples show how monitoring the self and keeping an eye on how identified risks develop, are one type of action triggered by DTC test results.

A rarer systemic consequence is described by an informant who used the test to prove that he did not have a previously identified inherited heart disease but instead suffered from severe stress that should be treated differently. This revelation of a misdiagnosis led to the removal of a heart implant, which the tester had received to treat erroneously diagnosed heart arrhythmia: ‘So it kind of sent me on a journey and it has taken me 10 years to get the doctors to admit that they made a mistake. That is to say, I was misdiagnosed. I never had that heart disease. I was diagnosed based on a false positive and have then used genetic tests to document that I do not have that heart disease’ (M40). In this example the DTC test result ended up having a demedicalising effect (Conrad 2007) as it spurred the tester to focus more on stress and lifestyle as the cause of his heart problems.

#### 4.2.2 Lifestyle Changes and Experimentation

Some informants describe how they have used the tests to either engage in new or confirm the value of already ongoing health activities. One informant explains: ‘I think I live a lot healthier. (…) It's like you can have a genetic risk and if you do good things the genetic risk will only be a little bit of a danger (…)’ (M35). Such reactions cannot always be seen as direct responses to a specific detected risk, but often also as general and commonsensical responses (e.g., to eat healthy, to exercise, to avoid smoking and drinking too much alcohol) to the knowledge that the tester has some sort of genetic disposition. Another informant explains that the test had consequences for her eating habits: ‘In terms of behavioural changes, I also know I have a gene that means my lipids can become imbalanced and that also makes me (…) think a bit more about how I eat, particularly in relation to the types of fats I consume’ (F45). She continues to explain that in a way the tests simply confirmed the value of well‐known health advice: ‘to eat vegetables, more vegetables, drink water and sleep well. Then it's really the same things we end up doing, you could say, regardless of what imbalances we might have’ (F45). According to the informant, the tests simply seem to motivate users to engage in health behaviour that could prolong life independent of which potential risk you are living with. A male informant insisted that the test had had an impact on his life but also that it was more related to confirming a preventive approach than pointing him towards very specific or new behavioural changes:

So, I think it's more a general orientation toward this preventive mindset. Everything I do professionally is very much about the nervous system – about balancing it. Because if we balance the nervous system, we can get ahead of chronic diseases –potentially delay them by 5, 10 or 15 years.

(M40)

Other informants stressed that genetic risk assessments are very hard to translate into specific actions, but instead of following general health advice they began experimenting with changes to measure their felt impact on life and well‐being. One informant explains how the vague actionability of test results spurred playing and tinkering instead of passivity:

But with these things often it's not clear how you should treat them and what the doses should be. It has not been studied well enough, so it is a lot of, I guess that's where the hacking idea comes in, there is a lot of tinkering and playing and sort of ‘Is this going to help? I'm not sure. Is it helping?’ It's really hard to – because I'm doing so many things all at the same time – so it becomes not scientific, you know. Because you don't have the time to run a placebo blind control on yourself. So, you just go: ‘Let's do this, let's do this, let's just try these things’. They are relatively low risk.

(M34)

Following this line of thinking test results become one of many different sources of knowledge that the biohacker tries to turn into activities with positive health outcomes but based on experimentation and a trial‐and‐error‐logic, not necessarily medically validated ideas of activities that directly addresses a certain genetic disposition.

#### 4.2.3 Epigenetic Knowledge Production

Some informants also stress that their engagement with genetics and its consequences is a life‐long process of reading, searching and finding knowledge—not a one‐off response. An informant explains that she often uses SNPedia, a site that she refers to as the encyclopaedia of genes, to learn about specific aspects of her genome (F63). Another type of consequence is connected to either processing the test results in new ways to make them more actionable or by engaging with other types of epigenetic tests to produce test results with more direct guidance. A male informant was asked if the test had had any direct consequences:

No, it has not. I don't really think it has. No, only that I am now just more receptive to the idea that having that information could be useful. So maybe if I get more information with a higher resolution, it maybe has more interesting insights. The genetic life hack website is much more interesting and more useful in decision making. Because there is stuff there on methylation and stuff like this (…).

(M34)

The website referred to—geneticlifehacks.com—is characterised by offering a different kind of analysis from the raw data of test results from for instance 23andMe or AncestryDNA. By repurposing the data through this third party tool the tester is offered suggestions on how the potential negative effects of genetic variants can be decreased through, for example, nutrition or supplements.

Other informants describe how they have left behind tests focused on polygenic risk in favour of epigenetic tests assessing biological age and methylation levels. This shifts the focus from stable genetic dispositions—which are often downplayed or normalised by the informants (‘we all have a little bit of cancer’, as one informant argued [M41])—in favour of measuring the body's functional age and cellular processes. This for many informants delivers better on their desire for genetic agency by outlining what can potentially be done/changed/affected (in more or less validated ways) instead of simply identifying what cannot in itself be changed (the existence of a certain genetic disposition).

#### 4.2.4 Social Implications

DTC GTs also produce new and complex genetic bonds. In that way, the test results have social consequences, which are often similar to those described in relation to other forms of (clinical) genetic testing. In line with existing research the informants describe an experience of having to navigate complex genetic ties with their relatives (Arribas‐Ayllon et al. 2008). This is expressed by the informants through reflections about how the genetic knowledge provided by the test can be used to care for their relatives through processes of sharing results and making new tests, but also about how relatives might want to get access to or avoid genetic knowledge in different ways.

A female informant very clearly describes that for her the use of DTC GT is a matter of caregiving, not of performance:

For me it's not performance or competition; it's something I do for myself, really. And in our family, by the way, because I've had genetic tests done for the whole family. Then I can ensure that we are aware of the risks we have, because we all have some risks – cancer, dementia, cholesterol and so on. Even my daughters and we might be able to talk about whether it's problematic to do genetic tests on children without they themselves have given you permission to do so, but I would like to make sure that I have complete control over allergies and their risks in relation to different things, cholesterol – we have cholesterol in our family and so on.

(F37)

The quote points to the blurred area between control and care, but also to how DTC GTs are not only used for individual purposes, but sometimes also to ensure the life and thriving of family members. Other informants articulate how the test—and the knowledge it produces—creates conversations and negotiations among their family members. One male informant (M35) plans to give his brother a test to clarify if he has a certain disposition, whereas another explains how his test led to his siblings and parents also taking the test (M43). In this latter case the test became the starting point for a family‐based investigation of which genetic dispositions are inherited and shared or not.

In other cases, the users' test results must be handled with more caution, because certain family members would prefer not to know about them. Especially the siblings of the informants are described as dilemmatic and genetically adjacent bodies in the sense that they are granted the right not to know, whereas the informants' own children are more often described as individuals that need to be tested or informed about risks as a part of the parental responsibilities of the informant. An informant describes how he introduced his family to the existence of the tests but got very mixed responses from his siblings: ‘I shared it with my wife and then I also tried to bring it up a bit with my family. (…) I think they were very mixed. My sister said: “Definitely, I would like that”. And my little brother was more like: “No, I wouldn't want to know, I'd rather remain in the dark”’ (M34).

Following this, DTC GTs are perceived by many informants as involved in various caregiving practices where knowledge becomes a dilemmatic aspect of this care. Care could thus involve making new tests of family members, starting conversations about the test results and the potential of buying genetic tests, but also allowing family members the right not to know about their genetic risks.

## 5 Discussion

By exploring the actionability work of biohackers in relation of DTC GTs, the article (1) produces new knowledge on biohackers' engagement with and reasons for using, a particular form for genetic technology and (2) contests the claim made in previous research on genetic testing that DTC GT results are not acted on or involved in behavioural change. By zooming in on biohacking, the article advances our understanding of genetic testing by highlighting both the direct consequences of getting knowledge about specific risks, like targeted lifestyle actions or self‐surveillance practices and the more indirect implications of testing, like re‐purposing data through third party tools or moving on to other technologies that are experienced as more directly actionable.

Ad (1) The article contributes to studies of biohacking activities with its focus on the deployment of a particular technology aimed at genetic knowledge production outside health institutions. The informants are quite diverse in their understanding of what biohacking is, ranging from more atypical practices of, for example, micro‐dosing or use of anti‐radiation technology to mainstream food supplements. The study supports existing research showing how biohackers engage with a variety of knowledge forms to engage in prevention and self‐optimisation (Cozza et al. 2022; Delfanti 2012). It also adds to this research by offering a conceptual understanding, using the notion of the milieu, of how biohacking approaches the body as deeply entangled with its surroundings but also by treating these surroundings as a medium for producing a desired future trajectory for the biohacking body. As the analysis shows, this desire for control is intertwined with a particular type of genetic imaginary, which is explicitly articulated by informants as ‘epigenetic’. This implies that a negative genetic risk assessment is understood as a chance to positively affect or shape the body by re‐arranging its milieu. Epigenetics is used by the informants to enable a sense of agency, but it also produces a tension as it is not always clear how to address the genetic dispositions revealed by the test through manipulations of the milieu. The analysis identifies both how biohacking engages with genetic technologies to enhance control and genetic agency and how biohacking subjects are not always sure about what should be done to enhance agency through the use of DTC GTs.

The study also contributes to the research on biohacking by stressing how its engagement with genetic knowledge production is often intertwined with a critical approach to healthcare and its perceived lack of interest in prevention through genetics. This critical approach is triggered by personal experiences of suboptimal care or of a more general preoccupation with advancing and optimising health care. Returning to the three archetypes of biohackers represented by Rifai—the citizen scientist, the cyborg and the superhuman (Rifai 2022)—the informants primarily position themselves as individuals in quest of wellbeing, slow and healthy ageing (cf. the superhuman position), but they have traits in common with the citizen scientist that performs scientific activities outside institutions. The cyborg, however, is by many informants associated with a public image of biohacking that they wish to detach themselves from as it is understood as stigmatising and too extreme. In other words, the informants do not show any interest in creating human‐machine‐hybrids or in the use of implants, but they resemble both the citizen scientist due to their critique of the established healthcare system (but not of research, which they generally seem to respect) and the type of biohacker focused on life optimisation and biological enhancement.

Ad (2) As mentioned, studies of users suggest a diversity of motivations for engaging with DTC GTs (Gerdes et al. 2021; Roberts et al. 2017), but the behavioural impact of such tests have been shown to be minimal (McGowan et al. 2010) and users often do not ‘act on results’ (Nolan and Ormondroyd 2023, 18). The novelty of the case of biohacking for understanding DTC genetic tests is that biohackers express a strong dedication to translate a range of knowledge forms into action and for that reason allow us an initial understanding of some of the unexplored ‘actionability work’ involved in using DTC GTs. The article thus adds nuance to existing work on DTC GTs by showing how a group of users, drawing on an epigenetic line of thinking, bridge the gap between results and action in more or less experimental ways. The analysis, however, does not show how general users act on DTC GT results, but rather the types of action that a particular group of agency‐seeking and milieu‐shaping users find relevant after getting the results.

More specifically, the actions and consequences of using DTC GTs involve (1) systemic or self‐initiated surveillance or correction of identified risk, (2) lifestyle change and experimentation perceived as either targeting identified risks or as broad responses that will likely have a positive effect on health, (3) repurposing raw test data for epigenetic analysis via third party tools or moving on to the market of epigenetic tests due to the perceived lack of actionability of DTC GTs and (4) various social and relational consequences as individual test results must be handled, shared with—or kept from—relatives. By outlining these types of action, the article advances research by showing how DTC GTs do become the starting point for various reactions and consequences among a particular user group.

In including the latter social category, the article does not only address traditional health‐related consequences, but also implications of a more relational kind. Looking at research on genetic test experiences that predate DTC GTs, it is important to stress that DTC GTs are in no way unique in their ability to stir up complex family stories, dilemmas and negotiations. This is also the case in relation to more clinically based genetic testing and counselling practices (Kenen et al. 2003; Arribas‐Ayllon et al. 2008; Svendsen 2002; Youssufi and Huniche 2017). What is new is rather that these processes are increasingly ‘deinstitutionalised’ and enacted in private settings disconnected from medical and counselling. The experienced relational consequences of this difference in access to guidance calls for more research.

Limitations of the study are its relatively small sample of informants and its engagement with a group of test users with a high interest in health and with substantial economic and educational resources. It is therefore difficult to determine if biohackers provide a potential image of how users in the future will engage with DTC GT and if more users will engage with this type of knowledge production. Gerdes et al. dare to make a guess regarding the latter question, as they state that an:

increase in the use of DTC‐GT is to be expected, as the awareness of the possibilities of online genetic testing in the population is increasing in combination with the empowerment of patients and citizens as part of the strategy for personalized medicine.

(Gerdes et al. 2021, 857)

If this is the case, more people will be left with the challenge of what to do with knowledge about their genetic risk and of trying to make sense of what—if anything—can be done with it. Biohackers may provide a preliminary understanding of some of the dilemmas and potential roads to action that these users will encounter and explore.

## 6 Conclusion

The article has investigated how a particular user group explores the actionability of an increasingly accessible type of genetic knowledge technology. The analysis shows how biohackers do not treat genetic risk assessments as a dreaded verdict. Rather they understand them as chances to positively affect or shape the body and its future. They use the concept of epigenetics to validate this sense of and search for genetic agency, but this notion also produces a tension as it is not always clear what turns gene expression on/off or what to do with the genetic risks revealed by DTC test results. The analysis identifies four different types of action enacted by the informants and in that way contributes to the academic understanding of how DTC test results are turned into action by a particular group of users in search of genetic agency. The actionability work of the informants points to a cultural situation where knowledge of genetic risk is increasingly accessible, but often also hard to act on by users. This is not least the case for those less interested in acquiring new health knowledge and less willing to experiment than the informants in this study.

## Author Contributions

**Carsten Stage:** conceptualization, investigation, funding acquisition, writing – original draft, methodology, validation, writing – review and editing, project administration.

## Funding

The article is based on research supported by a grant by the Independent Research Fund Denmark (IRDF, ID: 10.46540/2027‐00015B).

## Ethics Statement

According to Danish standards, the type of data collection reported in the manuscript is considered exempt from institutional ethical approval. Following the ethical provisions of the Danish National Center for Ethics, approval by an ethics committee is not required for interview‐based studies that do not involve human biological material. Nevertheless, the research was conducted in accordance with general ethical standards for good qualitative research. All participants provided written informed consent at the beginning of the interview and were informed of their right to withdraw participation and to review their interview transcripts prior to analysis (see Methods section). Data were handled in compliance with GDPR.

## Conflicts of Interest

The author declares no conflicts of interest.

## Data Availability

Research data are not shared.
